# Reduced Intensity Conditioning, Combined Transplantation of Haploidentical Hematopoietic Stem Cells and Mesenchymal Stem Cells in Patients with Severe Aplastic Anemia

**DOI:** 10.1371/journal.pone.0089666

**Published:** 2014-03-03

**Authors:** Xiao-Hong Li, Chun-Ji Gao, Wan-Ming Da, Yong-Bin Cao, Zhi-Hong Wang, Li-Xin Xu, Ya-Mei Wu, Bei Liu, Zhou-Yang Liu, Bei Yan, Song-Wei Li, Xue-Liang Yang, Xiao-Xiong Wu, Zhong-Chao Han

**Affiliations:** 1 Department of Hematology, The First Affiliated Hospital, Chinese PLA General Hospital, Beijing, China; 2 Department of Hematology, Chinese PLA General Hospital, Beijing, China; 3 TEDA Research Center of Life Science and Technology, State Key Laboratory of Experimental Hematology, Institute of Hematology, Tianjin, China; Wake Forest Institute for Regenerative Medicine, United States of America

## Abstract

We examined if transplantation of combined haploidentical hematopoietic stem cells (HSC) and mesenchymal stem cells (MSC) affected graft failure and graft-versus-host disease (GVHD) in patients with severe aplastic anemia (SAA). Patients with SAA-I (N = 17) received haploidentical HSCT plus MSC infusion. Stem cell grafts used a combination of granulocyte colony-stimulating factor (G-CSF)-primed bone marrow and G-CSF-mobilized peripheral blood stem cells of haploidentical donors and the culture-expanded third-party donor-derived umbilical cord MSCs (UC-MSCs), respectively. Reduced intensity conditioning consisted of fludarabine (30 mg/m^2^·d)+cyclosphamide (500 mg/m^2^·d)+anti-human thymocyte IgG. Transplant recipients also received cyclosporin A, mycophenolatemofetil, and CD25 monoclonal antibody. A total of 16 patients achieved hematopoietic reconstitution. The median mononuclear cell and CD34 count was 9.3×10^8^/kg and 4.5×10^6^/kg. Median time to ANC was >0.5×10^9^/L and PLT count >20×10^9^/L were 12 and 14 days, respectively. Grade III-IV acute GVHD was seen in 23.5% of the cases, while moderate and severe chronic GVHD were seen in 14.2% of the cases. The 3-month and 6-month survival rates for all patients were 88.2% and 76.5%, respectively; mean survival time was 56.5 months. Combined transplantation of haploidentical HSCs and MSCs on SAA without an HLA-identical sibling donor was safe, effectively reduced the incidence of severe GVHD, and improved patient survival.

## Introduction

Severe aplastic anemia (SAA) is a life-threatening disorder which is currently treated by allogeneic hematopoietic stem cell transplantation (HSCT), which is based on full human leukocyte antigen (HLA) compatibility between the donor and patient. However, patients who do not find a matched donor can benefit from immunosuppressive therapy or HSCT from an unrelated donor. Haploidentical related transplantation is not limited by the source of stem cells, but rather by the high incidence of transplantation failure and refractory graft-versus-host disease (GVHD) [1].

Cyclosphamide (CTX)-based conditioning has been used as an immunosuppressive therapy for HSCT with a matched related donor [2]. Since transplantation with a matched unrelated donor is associated with a high incidence of rejection, this conditioning regimen is not sufficient [3]. Mixed results have been reported by a number of groups which have investigated methods to increase the immunosuppressive activity of the conditioning regimen, including total body irradiation to CTX or using a combination of fludarabine, CTX, and antithymocyte globulin [4], [5]. It has recently been suggested that a reduced dose of CTX may offer benefits (ie, reduced side effects) and improve overall prognosis [6], [7].

Mesenchymal stem cells (MSCs) represent a type of adult stem cells found in multiple tissues and organs, with potential for self-renewal and multi-lineage differentiation. MSCs also have the ability to regulate immune function. MSCs act as precursor cells in the bone marrow stroma and MSCs isolated from bone marrow, blood, adipose tissue, fetal tissue and cord blood were shown to enhance engraftment after hematopoietic stem cell transplantation and to facilitate engraftment of neutrophils and platelets and promote 100% donor chimerism [8], [9]. Infusion of MSCs has also been demonstrated to reduce the incidence of both acute and chronic GVHD [9].

Previous studies also showed that 1) peripheral blood HSCT aided rapid recovery of hematopoietic function, and reduced rate of infection, 2) G-CSF mobilized bone marrow and peripheral blood are an enriched source of stem cells which can be used to facilitate the implantation of allogeneic HSCs, and reduce the incidence of GVHD, and 3) umbilical cord MSCs increase the implantation rate, are readily available, and have a potent proliferative capacity [10], [11]. Based on these findings, we used a combination of granulocyte colony-stimulating factor (G-CSF)-primed bone marrow and G-CSF-mobilized peripheral blood stem cells of haploidentical donors and culture-expanded third-party donor-derived umbilical cord MSCs (UC-MSCs) along with a modified regimen of conditioning. We evaluated GVHD and survival in 17 SAA patients subjected to this modified regimen.

## Materials and Methods

### Subjects

In this retrospective study, we enrolled a total of 17 patients who were diagnosed with severe AA (SAA) according to the SAA criteria defined by the Criteria for Diagnosis and Treatment of Hematological Diseases, between October 2006 to October 2012 [12]. All 17 study subjects were eligible for this study based on the following inclusion criteria: (1) Diagnosed with SAA or VSAA, as defined by the International Aplastic Anemia Study Group; (2) Lack of response to previous therapy, including CsAþstanozole/andriol ± G-CSF ± anti-human thymocyte IgG (ATG) ± EPO ± glucocorticoid; (3) Recipients of multiple transfusions and transfusion dependent at the time of transplantation; (4) Voluntary participation in HSCT; (5) Absence of uncontrolled infections and severe liver, renal, lung and heart diseases; (6) Lack of available, HLA-identical, related sibling or unrelated donor; and (7) Written informed consent obtained from patients or their guardians and donors. Patients were contacted by phone every three months for 3 years as part of a long-term follow-up. This study was approved by the Institutional Review Board of the General Hospital of Chinese People's Liberation Army.

### HLA-partially matched donors

Peripheral blood (5 ml) was collected via the cubital vein and anti-coagulated with sodium citrate. Peripheral white blood cell counts were <0.5×10^9^/L; bone marrow (5 ml) was collected from the sternum. Total DNA was extracted using the QIAamp Blood Kit (Qiagen) according to the manufacturer's instructions. Site A of HLA-I was detected using a PCR-SSP low resolution kit (PEL-FREEZ) and amplified using a thermal cycler (PE-9700). The products were then subjected to 2% agarose gel electrophoresis for further analysis.

### Regimen for reduced conditioning

The conditioning regimen included cyclophosphamide (CTX): 500 mg/(m^2^·d), rabbit anti-human lymphocyte globulin (ATG): 5 mg/(kg·d), Fludarabine (Flu): 30 mg/(m^2^·d). Conditioning was done intravenously for 4 consecutive days. Additionally, for prevention of GVHD, intravenous Cyclosporine A (CsA; 3 mg/kg/day) was administered in divided doses beginning on the day before transplantation (Day −5) and was continued throughout the transplantation period. Patients were advanced to oral cyclosporine as tolerated. In the absence of GVHD, the oral CsA dose was reduced weekly by approximately 5% beginning on or near 6 months, and therapy was usually discontinued by one year after transplantation. Mycophenolate mofetil (MMF) was administered orally at a dose of 500 mg/day starting on day 3 before transplantation and was tapered off after 90 days if no aGVHD was observed. CD25 monoclonal antibody (20 mg) was administered once on day 4 after transplantation, and used thereafter only if GVHD was indicated.

### Collection of stem cells

**Figure 1:**
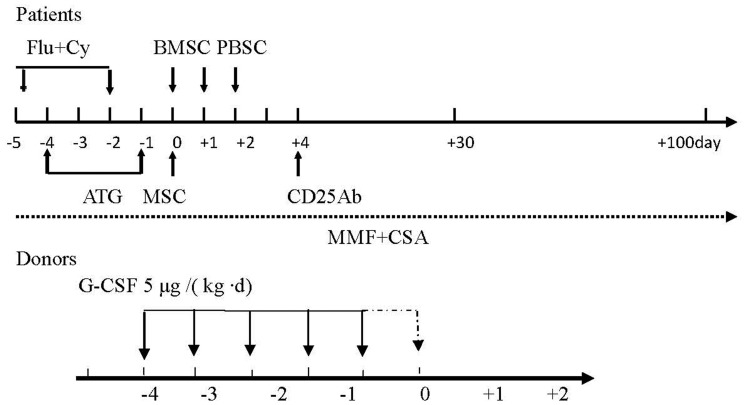
Graphic representation of conditioning regimen.

All donors were subcutaneously treated with 5 µg G-CSF/(kg·d) for 5–6 consecutive days. A total of 200–500 ml of bone marrow was collected (3–4×10^8^/kg nucleated cells) at the posterior superior iliac ridge 4 hours after injection on day 5, and transfused into the patient. This corresponded to Day 0 in the recipient cycle (Figure 1). For patients with major ABO incompatibility, hydroxyethyl starch was used for red blood cell sedimentation after collection of bone marrow. On days 6 and 7, peripheral stem cells were separated using CS 3000 plus Blood Cell Separator (Baxter) to a circulating volume of 10 L and a final volume of 0.055 L. The stem cells were then counted and immediately transfused into patients. This corresponded to Days 1 and 2 in the recipient cycle (Figure 1). The mean number of transfused mononuclear cells (MNC) was 9.34×10^8^/kg (6.3–13.2)×10^8^/kg, and the mean number of CD34+ cells was 4.5 (2.8–8)×10^6^/kg.

### Mesenchymal stem cells preparation and transfusion

UC-MSCs were purchased from the National Engineering Research Center of Cell Products, State Key Laboratory of Experimental Hematology. The UC-MSCs were positive for CD13, CD29, CD90, CD44, CD105 (SH2), CD106, CD73 (SH3), CD166, and HLA-ABC, and negative for CD14, CD34, CD38, CD45, CD31, and HLA-DR. CD106 and HLA-ABC were expressed at significantly lower levels than the other markers, and each patient received UC-MSCs from a single donor, as previously described [13]. The UC-MSCs were collected and prepared on Day 0 and used immediately as a fresh preparation. Transfusion recipients received the human umbilical cord-derived mesenchymal stem cells (hUC-MSC; 4 [2.87–10]×10^6^/kg) 6 hours before transfusion of bone marrow and peripheral blood stem cells (Figure 1). Patients were treated with sodium bicarbonate, dexamethasone and promethazine before transfusion, and simultaneously subjected to treatment for anti-inflammation, hepatoprotection and protection of gastric mucosa (2 mg Dexamethasone, Sodium hydrogen carbonate, Omeprazole). Patients were monitored for vital signs and symptoms of allergy during the transfusion, as well as at 30 minutes, 4 hours, 24 hours and 72 hours post transfusion.

### Prevention of graft versus host disease

Immunosuppressive agents included CsA, MMF, anti-thymocyte globulin (ATG), and CD25 monoclonal antibody (basiliximab monoclonal antibody; Novartis, Switzerland). CsA was administered 5 days before transplantation (3 mg/kg). Trough concentration of CsA was monitored using a fluorescence polarization immunoassay, a target trough blood concentration of 150–250 ng/ml was requested till 12 months after HSCT and was gradually tapered thereafter. CsA was completely withdrawn in the next 2–3 months. MMF (500 mg/d) was administered 3 days before transplantation and was discontinued 90 days after transplantation. CD25 monoclonal antibody (20 mg) was administered 4 days before transplantation; thereafter, CD25 antibody was used only if GVHD was indicated.

### Prevention of other complications

General preventive measures were performed 1–2 days before conditioning. Patients had a bath with enema drug and the skin was prepared routinely. Patients stayed in a laminar flow clean ward and were treated with acyclovir, ornidazole, and sulfamethoxazole trimethoprim until 1 day before transplantation. Three days after transplantation, rhG-CSF and TPO were injected subcutaneously until hematopoietic function was recovered. A blood transfusion was performed once hemoglobin <60 g/L and/or platelets <15×10^9^/L. All the blood products underwent ^60^Co radiation. For patients with hepatic veno-occlusive syndrome, prostaglandin E (PGE1; 20 µg/d) and salvia (20 mg/d) were administered via IV from the day of conditioning to 14 days after transplantation.

For prevention of hemorrhagic cystitis, 2-mercapto-ethyl sulfonate (Mesna) was used (1.3 fold of CTX dose) at 0, 4 and 8 hours after CTX treatment. Hydration and alkalization of urine were performed simultaneously. A diuretic (furosemide) was administered intravenously with a daily urine volume assured to be >80 ml/kg. For prevention of cytomegalovirus infection, patients were treated with either ganciclovir (10 mg/(kg·d) or foscarnet (3–6 g/d) for 14 days until 1 day before transplantation. After transplantation, patients were tested for CMV-DNA and EBV-DNA every week, and anti-viral treatment was applied according to the results.

Patients were monitored weekly for CMV DNA (using real time PCR) and EBV-DNA after HSCT. Patients with CMV antigenemia received preemptive ganciclovir (DHPG) or foscarnet therapy. Human Ig (10 g) from healthy volunteer donors was intravenously administered each week during the first month.

### Evaluation of implantation and observations

Myeloid engraftment was defined as the first of three consecutive days with an ANC 0.5×10^9^/L, and platelet engraftment was defined as the day the platelet count met or exceeded 20×10^9^/L without transfusion for a week. Hematopoietic chimerism was evaluated by PCR amplification of STRs for sex-matched pairs using peripheral blood samples from the donor and the recipient. After HSCT, recipient BM samples were drawn monthly for the first 3 months and every 3–6 months thereafter for an additional 1–2 years. Complete donor chimerism was defined as the presence of only donor-type hematopoietic cells after allogeneic BMT. Primary graft failure was defined by the absence of hematological recovery in patients surviving 21 days after transplantation, and late rejection was defined as graft loss after initial graft function, that is, complete or partial recovery of hematopoiesis of donor origin followed by recurrent pancytopenia with a markedly hypocellular BM in the absence of moderate to severe acute GVHD [10], [11]. The incidence and severity of GVHD were based on guidelines from an NIH consensus conference to determine GVHD grade [14]. Patients were evaluated for cGVHD if they survived for at least 100 days after HSCT.

Thirty days after transplantation, peripheral blood was collected and chimera was detected in the Huada Fangrui Evidence Identification Center of Justice.

### Statistical analysis

Continuous data were presented as median (range) due to the overall small sample size. Categorical data were presented as count and percentage. The overall survival rate was evaluated using the Kaplan-Meier estimate. The incidence of acute and chronic GVHD was evaluated by the Kaplan-Meier estimate of disease-free rate. Two-tailed p-values less than 0.05 were considered statistically significant. Statistical analyses were performed using SPSS 15.0 (SPSS Inc, Chicago, IL, USA).

## Results

### Patients and their donors

A total of 17 patients (7 women and 10 men) who were initially diagnosed with SAA were enrolled in the study. The patients were all aged less than 30 years, with a median age of 19 years (range: 4–29 years). There were no HLA-identical related donors and unrelated donors, and all patients were subjected to haploidentical related HSCT. HLA-A, B, and DRB1 incompatibility was observed in 12 patients, while HLA B and DRB1 incompatibility was observed in 2 patients and HLA-A, B or A, DRB1 or A in 1 patient (12 out of 17 pairs had 3-loci mismatched; 4 out of 17 pairs had 2-loci mismatched). Additional patient characteristics are shown in **Table 1**.

**Table 1:** The characteristics of 17 patients treated with haploidentical HSCT.

		N = 17
Gender	Female	7 (41.2%)
	Male	10 (58.8%)
Age (year)		19.0 (4.0, 29.0)
Time from diagnosis to transplantation (month)		3.0 (1.0, 5.0)
Transfusion RBC before HSCT (U)		12.0 (4.0, 22.0)
Transfusion PLT before HSCT (U)		9.0 (3.0, 16.0)
Disease and status at transplantation	SAA	8 (47.1%)
	VSAA	9 (52.9%)
Patient blood type	A	6 (35.3%)
	AB	2 (11.8%)
	B	6 (35.3%)
	O	3 (17.6%)
Donor's age (year)		38.0 (16.0, 49.0)
Donor's gender	Female	10 (58.8%)
	Male	7 (41.2%)
Donor-recipient relationship	brother-brother	2 (11.8%)
	father-daughter	2 (11.8%)
	father-son	3 (17.6%)
	mother-daughter	2 (11.8%)
	mother-son	4 (23.5%)
	sister-brother	1 (5.9%)
	sister-sister	3 (17.6%)
Donor's blood type	A	7 (41.2%)
	B	5 (29.4%)
	O	5 (29.4%)
Gender match (donor-recipient)	female-female	5 (29.4%)
	female-male	4 (23.5%)
	female-man	1 (5.9%)
	male-female	2 (11.8%)
	male-male	5 (29.4%)
Blood type match (donor-recipient)	A-A	6 (35.3%)
	A-B	1 (5.9%)
	B-AB	1 (5.9%)
	B-B	4 (23.5%)
	O-AB	1 (5.9%)
	O-B	1 (5.9%)
	O-O	3 (17.6%)
HLA match (donor-recipient)	A	1 (5.9%)
	A,B	1 (5.9%)
	A,B,DRB1	12 (70.6%)
	A,DRB1	1 (5.9%)
	B,DRB1	2 (11.8%)
CMV donor/recipient serostutus	+/−	1 (5.9%)
	+/+	16 (94.1%)
Stem cells		
Mononuclear cells (10^8^/kg)		9.3 (6.3, 13.2)
CD34+ count (10^6^/kg)		4.5 (2.8, 7.9)
Time of ANC >0.5×10^9^/L (day)		12.0 (11.0, 21.0)
Time of PLT count >20×10^9^/L (day)		14.0 (11.0, 75.0)

The median time from diagnosis to transplantation was 3 months (range: 1–5 months). Nine out of 17 patients were considered to have VSAA; the remaining 8 patients were considered to have SAA. All donors tolerated the procedures well, and no severe side effects occurred during BM harvest and leukapheresis. The median mononuclear cell and CD34 counts were 9.3×10^8^/kg and 4.5×10^6^/kg, respectively. Median time to ANC was >0.5×10^9^/L and PLT count >20×10^9^/L were 12 and 14 days, respectively.

### Engraftment

Myeloid recovery was achieved in all 17 patients after transplantation without primary graft failure. Sixteen patients (94%) achieved full donor chimerism within 30 days after HSCT. A single patient (1 out of 17 [6%]) had transient engraftment and developed late graft failure on day 37 after transplantation and was treated with CTX followed by an infusion of PBSCs from the original donor on day 59. Following this infusion, the patient died on day 75 after transplantation, and platelet engraftment was not achieved before death.

### Acute GVHD

**Figure 2:**
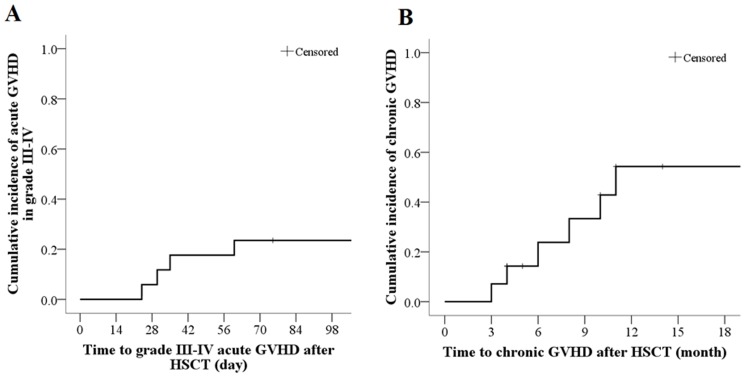
The Kaplan-Meier curve for the cumulative incidences of grade III–IV acute GVHD (A) and chronic GVHD (B).

Acute GVHD was not observed in 7 out of 17 patients (41.2%), 3 patients (17.6%) had grade I GVHD, 3 patients (17.6%) had grade II GVHD, and 4 patients (23.5%) had grade III–IV acute GVHD. Patients with III–IV aGVHD received pulse therapy with methylprednisolone (2 mg/kg/d for 5 days) and CD25 monoclonal antibody. Three of these patients showed improvement. Eight weeks after transplantation (56 days), the cumulative incidence of acute GVHD grades III–IV was 17.6% (3 out of 17 patients); and the last acute GVHD grades III–IV was observed 60 days after transplantation (**Table 2** **,** **Figure 2A**).

**Table 2:** Graft, acute GVHD, chronic GVHD, infection of virus and causes of death after haploidentical HSCT.

		N = 17
Graft failure		1 (5.9%)
Acute GVHD	None	7 (41.2%)
	Grade I	3 (17.6%)
	Grade II	3 (17.6%)
	Grade III	2 (11.8%)
	Grade IV	2 (11.8%)
Chronic GVHD*	None	8/14 (57.1%)
	Mild	4/14 (28.6%)
	Moderate	1/14 (7.1%)
	Severe	1/14 (7.1%)
Viremia		
CMV		7 (41.2%)
EBV		3 (17.6%)
Non-infection complication		
VOD		0
Haemorrhagic cystitis (grade II)		2 (11.8%)
Death		5 (29.4%)
Cause of death		
Late rejection		1 (5.9%)
GVHD		2 (11.8%)
Infection		2 (11.8%)
Follow-up time (month)		10.0 (2.5, 80.0)

*14 patients were survived more than 100 days after transplantation.

### Chronic GVHD

Of a total of 14 patients who survived for more than 100 days after transplantation, 6 patients (42.8%) developed chronic GVHD. Four of the 14 patients (28.6%) showed mild chronic GVHD, 1 patient (7.1%) showed moderate chronic GVHD, and 1 patient (7.1%) showed severe chronic GVHD. The 6-month cumulative incidence of chronic GVHD was 42.8%. The last chronic GVHD was occurred in 11 months after transplantation (**Table 2** **,** **Figure 2B**).

### Complications

Overall, side effects were not observed after transfusion (**Table 2**). Nine of the 17 patients developed varying degrees of fever associated with hematopoietic dysfunction, which was resolved after treatment with anti-inflammatory medication. One patient died of sepsis 120 days after transplantation. Seven patients developed CMV infection, and infection with EB virus was observed in 3 patients, of whom 1 developed infection of CMV and EBV and died of viral infection 90 days after transplantation. Any viral infection in the remaining patients was treated with ganciclovir, or foscarnet and gamma globulin and all patients demonstrated a full recovery. Hemorrhagic cystitis was observed in 2 patients at 50 days and 43 days after transplantation, respectively. After hydration, alkalization of urine, and bladder flushing, both recovered within 2–3 weeks. VOD was not observed in this patient group.

### Overall survival and treatment-related mortality

**Figure 3:**
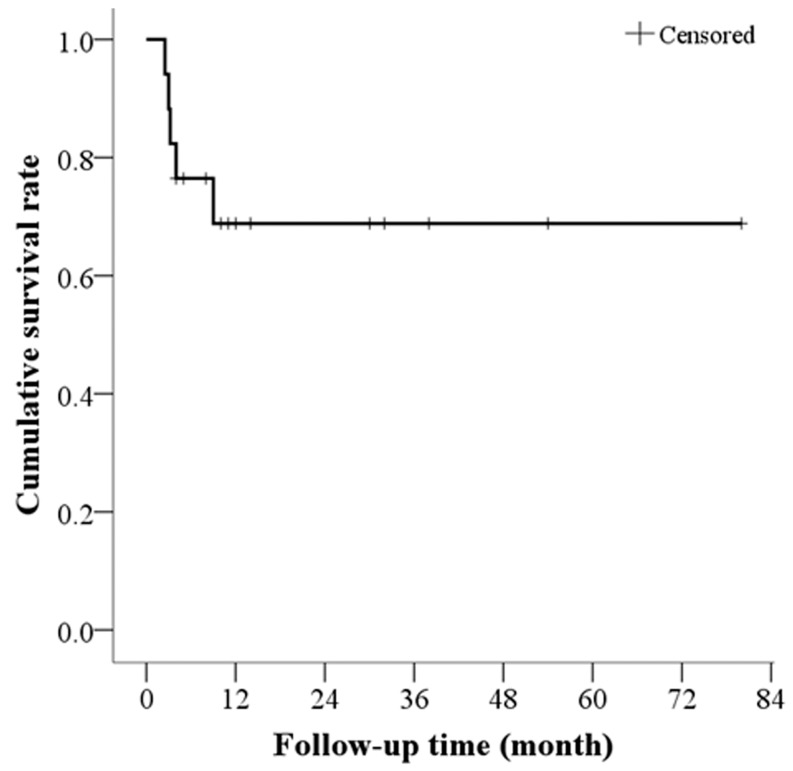
Cumulative survival rate (Kaplan-Meier).

Patients were followed-up for a median of 10 months (range 2.5–80 months). None of the patients were lost to follow-up. Five patients died within the follow-up period. The causes of death included GVHD (2 patients), infection (2 patients), and graft failure (1 patient). The 3-month and 6-month survival rate for all patients was 88.2% and 76.5%, respectively. The mean survival time was 56.5 months (**Figure 3**). All 12 of the surviving patients (beyond 9 months) achieved a hematologic CR.

## Discussion

Lack of timely and effective treatment for SAA patients can result in death due to infection and hemorrhage [12]. Since the key considerations when using HCST to treat SAA are 1) immunosuppression (conditioning), 2) the source of stem cells, and 3) prevention of GVHD, we modified a pre-existing regimen for conditioning, improved the source of stem cells, and took measures to prevent GVHD. We showed an overall survival rate of 70.58% within a median 2-year follow-up period in SAA patients after related-haploidentical HSCT in combination with umbilical cord blood mesenchymal stem cell transplantation.

Conditioning in AA patients is used to suppress immune function and inhibit rejection of donated HSCs. In addition to CTX and ATG, which are widely used for conditioning in AA patients [15], fludarabine (Flu), a cytotoxic immunosuppressant, has also shown efficacy during allogeneic transplantation in SAA patients, transplantation from unrelated donors [3], [5], [16], and in reduced intensity conditioning protocols [17]. In this study, patients received 30% of the standard CTX dose, and exhibited efficient implantation with few side effects and few conditioning-related complications.

MSCs in HSCT may promote the implantation of stem cells, improve the recovery of hematopoietic function and prevent GVHD. Haplo-HCST in combination with donor-derived umbilical cord MSCs was previously shown to be safe and feasible and decreased the severity of GVHD in patients with hematological malignancies [18]. In the present study, stem cells were collected from the bone marrow and peripheral blood of G-CSF-treated donors and transplanted in combination with umbilical cord blood MSCs. Although peripheral blood HSCT has the advantage of rapid recovery of hematopoietic function, reduced rate of infection, and early mortality, bone marrow is also a rich source of MSCs, which can be used to increase the implantation rate and reduce the incidence of GVHD [19]. Additionally, G-CSF mobilized bone marrow and peripheral blood have been reported as an enriched source of stem cells, with a low proportion of T cells, which further facilitates the implantation of allogeneic HSCs, and reduces the incidence of GVHD [10]. Interestingly, G-CSF-primed bone marrow and peripheral blood have previously been shown to contain lower proportions of T-cells compared to unstimulated controls. Peripheral CD34+ cell counts have been shown to peak 5 days after G-CSF administration [20]. Healthy donors who received 10 µg/kg of G-CSF for 2 days before harvest had similar numbers of nuclear cells, CD34+ cells, and CD3+ cells, but an increased number of granulocyte macrophage colony-forming units in the bone marrow compared to unstimulated bone marrow controls [21]. Additionally, mouse bone marrow cells collected 14 days after in-vivo administration of G-CSF and stem cell factors had a 10-fold greater ability to repopulate compared to untreated bone marrow [22]. These studies suggested that it was possible to obtain grafts with different cell distributions and immunogenetic characteristics by changing the proportions of G-CSF- primed bone marrow with G-PBSCs, which would result in larger numbers of stem cells, reduced GVHD, and rapid engraftment of allogeneic stem cells with relatively lower T- cell counts compared to using G-PBSCs alone. We also used umbilical cord MSCs, which are readily available, have a low possibility of viral infection, and potent proliferative capacity [11]. Co-culture of MSCs and allogeneic T cells failed to significantly increase the proliferation of allogeneic T cells in-vitro [14], suggesting that MSC transplantation is not limited by HLA matching due to low immunogenicity of MSCs.

Besides implantation failure, GVHD and infection are the major causes of death in SAA patients receiving haploidentical HSCT. Most transplantation centers remove T-lymphocytes in an effort to reduce the incidence of GVHD, although this also significantly increases the incidence of viral infection. In the present study, T cells were not removed, and GVHD prevention measures included modification of ATG and Flu regimens, reduction in the dose of MMF, CsA, and CTX, and administration of CD25 monoclonal antibody 4 days prior to transplantation. This regimen was previously shown to prevent GVHD after allogeneic haploidentical HSCT [23]. Our data suggested that MSCs attenuated GVHD. The risk of GVHD in our study was comparable or relatively lower than the risk reported in the literature, and only one of our study patients had severe GVHD resulting in death.

It is not clear if the hemorrhagic cystitis in our study patients was viral in nature. We did not investigate the presence of BK virus in our subjects since BK virus positivity is very high, and almost all patients with or without hemorrhagic cystitis are positive for BK virus. However, all our patients with hemorrhagic cystitis improved after treatment. Despite the lack of control subjects in this study, our survival data were encouraging and supported previous studies showing that haploidentical allo-HSCT combined with umbilical cord MSC infusion could be an effective approach to treat SAA [18], [24].

Sibling HLA identical HSCT is the preferred modality to treat SAA patients younger than 40 years [25]. However, there are very few HLA identical siblings, especially in China. Our data suggest that haploidentical HCST is an attractive option for SAA/VSAA patients who have no HLA-identical sibling or unrelated donors. Our data are particularly relevant since current practice guidelines do not recommend transplantation from an alternative donor as a first-line treatment. Although our present study showed the benefit of haploidentical HCST in patients <40 years old, we previously reported that 1) the oldest leukemia patient who underwent haplo-HSCT combined with third-party donor-derived UC-MSCs at our center was 58 years old, and 2) the oldest SAA patient who underwent haplo-HSCT combined with third-party donor-derived UC-MSCs was 31 years old, while the donor was 57 years old.

[18]. Further research is necessary to improve OS by decreasing GVHD and maintaining stable engraftment.
