# Correction: Reduced Intensity Conditioning, Combined Transplantation of Haploidentical Hematopoietic Stem Cells and Mesenchymal Stem Cells in Patients with Severe Aplastic Anemia

**DOI:** 10.1371/journal.pone.0117641

**Published:** 2015-02-12

**Authors:** The PLOS ONE Staff

There is an error in the fifth sentence of the “Mesenchymal stem cells preparation and transfusion” subsection of the Materials and Methods. The correct sentence is: Transfusion recipients received the human umbilical cord-derived mesenchymal stem cells (hUC-MSC; 4 [2.87–10]×10^5^/kg) 6 hours before transfusion of bone marrow and peripheral blood stem cells (Fig. 1).

## References

[1] Li X-H, Gao C-J, Da W-M, Cao Y-B, Wang Z-H, et al. (2014) Reduced Intensity Conditioning, Combined Transplantation of Haploidentical Hematopoietic Stem Cells and Mesenchymal Stem Cells in Patients with Severe Aplastic Anemia. PLoS ONE 9(3): e89666 doi: 10.1371/journal.pone.0089666 24594618 10.1371/journal.pone.0089666 PMC3940616

